# The TRPA1 Agonist, Methyl Syringate Suppresses Food Intake and Gastric Emptying

**DOI:** 10.1371/journal.pone.0071603

**Published:** 2013-08-21

**Authors:** Min Jung Kim, Hee Jin Son, Seo Hyeon Song, Myungji Jung, Yiseul Kim, Mee-Ra Rhyu

**Affiliations:** Division of Metabolism and Functionality Research, Korea Food Research Institute, Bundang-gu, Sungnam-si, Gyeonggi-do, South Korea; National Institute of Agronomic Research, France

## Abstract

Transient receptor potential channel ankryn 1 (TRPA1) expressed in the gastrointestinal tract is associated with gastric motility, gastric emptying, and food intake. In this study, we investigated the effects of methyl syringate, a specific and selective TRPA1 agonist, on food intake, gastric emptying, and gut hormone levels in imprinting control region (ICR) mice. The administration of methyl syringate suppressed cumulative food intake and gastric emptying. In addition, treatment with ruthenium red (RR), a general cation channel blocker, and HC-030031, a selective TRPA1 antagonist, inhibited methyl syringate-induced reduction of food intake and delayed gastric emptying in ICR mice. Methyl syringate also increased plasma peptide YY (PYY) levels, but not glucagon-like peptide-1 (GLP-1) levels. The elevation in PYY was blocked by treatment with RR and HC-030031. The present findings indicate that methyl syringate regulates food intake and gastric emptying through a TRPA1-mediated pathway and, by extension, can contribute to weight suppression.

## Introduction

The gastrointestinal (GI) tract, the largest endocrine organ, plays an important role in the regulation of energy homeostasis and gastric emptying by coordinating appetite, food intake, and body weight [1], [2]. Gastric emptying is promoted by increased food volume in the stomach, the presence of liquid food, and protein. In contrast, the osmolarity of chime, fat, duodenal distension, cold temperatures, and an acidic pH of 3.5–4 inhibit gastric emptying [3], [4]. Several GI hormones such as peptide YY (PYY) and glucagon-like peptide 1 (GLP-1) also regulate gastric emptying and food intake when secreted from L-cells in the ileum into the circulation after food consumption [5]. The effect of GLP-1 and PYY is late and long-term. GLP-1, found in the jejunum, ileum, and colon, is stimulated by carbohydrate, protein, lipid, and glucose concentration in blood [6]. GLP-1 release dose-dependently enhances insulin secretion from pancreas, insulin-sensitivity, and insulin gene expression [7]–[9]. GLP-1 also reduces glucagon secretion from the pancreas, gastric secretion, gastric emptying, and food intake. PYY increases electrolyte and water absorption in the colon, while suppressing pancreatic secretion, gastric motility, gastric emptying, and appetite [10], [11].

Transient receptor potential cation channel, subfamily A, member 1 (TRPA1), a member of the TRP family, is expressed in sensory neurons and associated with somatosensation, such as pain, cold, hot, and pungent irritants [12]–[15]. The recent identification of TRPA1 expression in other organs has revealed additional functions. TRPA1 is highly expressed in enteroendocrine cells in the GI tract of humans, mice, and rats, where it plays an important role in delayed gastric emptying, secretion of gut hormones and reduction of food intake [16], [17]. TRPA1 responds to various pungent ingredients in foods including allyl isothiocyanate (AITC) in wasabi, allicin in garlic, benzyl isothiocyanate in yellow mustard, isopropyl isothiocyanate in nasturtium seeds, methyl isothiocyanate in capers, phenylethyl isothiocyanate in Brussels sprouts, and ligustilide in celery (*Apium graveolens L.*) and lovage (*Levisticum officinale L*) [18]–[21]. Non-pungent compounds such as capsiate and the fatty acids in royal jelly are TRPA1 activators [22], [23].

We previously determined that methyl syringate is one of the pungent ingredients in *Kalopanax pictus* Nakai (*K. pictus*) [24]. Methyl syringate increases cytosolic Ca^2+^ in human TRPA1 (hTRPA1)-transfected cells. This effect is inhibited by a general cation channel blocker, ruthenium red (RR) or a selective TRPA1 blocker, HC-030031. Thus, methyl syringate is a specific and selective activator of hTRPA1. Methyl syringate may be an electrophile because it performs as an electon-transfer mediator in Bratkovskaya *et* *al.* (2006) [25]. The electrophilic TRPA1 agonists such as AITC and lots of α,β-unsaturated aldehydes are covalently modified cysteine residues in cytoplasmic domain of TRPA1 and activates TRPA1 [26]. Same as other electrophilic agonists, methyl syringate may activate TRPA1 via a covalent pathway. In this study, we investigated whether methyl syringate inhibits gastric emptying by activating TRPA1 and stimulates the release of gut hormones including PYY and GLP-1 *in vivo* in male imprinting control region (ICR) mice.

## Materials and Methods

### Reagents

**Figure 1:**
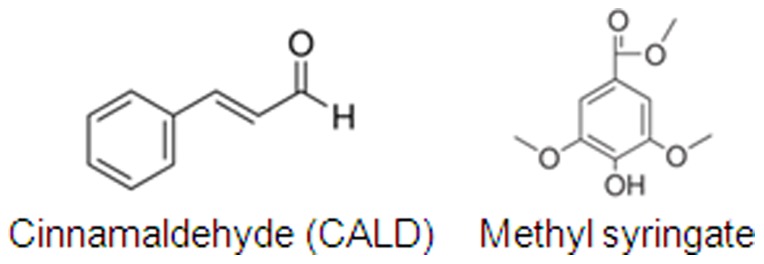
Chemical structures of cinnamaldehyde and methyl syringate.

Cinnamaldehyde, ruthenium red (RR), HC-030031, dimethyl sulfoxide (DMSO), phenol red, methyl cellulose, and urethane were purchased from Sigma-Aldrich (St. Louis, MO, USA). Trichloroacetic acid was obtained from Junsei Chemical Co., Ltd. (Chuo-ku, Tokyo) and methyl syringate from Alfa Aesar (MA, USA). The structures of cinnamaldehyde and methyl syringate were described in Figure 1.

### Animals

Male ICR mice (30–40 g) were obtained from Hanlim Laboratory Animal Co. (Gyeonggi-do, South Korea). Mice were housed in a room under a 12:12 h light: dark cycle (lights on 0700) with *ad libitum* access to standard laboratory chow and tap water. The room was maintained at 22±2°C with relative humidity at 59±1%. Prior to the experiments, all animals were housed in individual cages and fasted overnight with access to water at all times. The care and use of the animals followed our institutional and national guidelines, and the protocol was approved by the committee on the Ethics of Animal Experiments of the Korea Food Research Institute (Permit Number: KFRI-M-12028). All surgery was performed under sodium pentobarbital anesthesia, and all efforts were made to minimize suffering.

### Evaluation of food consumption and gastric resection

Mice were divided into treatment groups (n = 4 per group) and orally administered vehicle (1.5% methylcellulose) or 10 mg/kg cinnamaldehyde or methyl syringate by gavage. Immediately after treatment, the animals were given free access to their normal diet. Food intake was recorded 28 h after treatment. To investigate the effects of TRPA1 inhibition, 0.33 mg/kg RR or 0.15 mg/kg HC-030031 were administered with vehicle (1.5% methylcellulose) or 10 mg/kg cinnamaldehyde or methyl syringate. Food intake was recorded at 2, 4, 6, 8, 24, and 28 h after treatment. After 28 h, mice were sacrificed, the stomach of each mouse extracted, and contents weighed.

### Evaluation of gastric emptying

Gastric emptying was performed 5 min after treatment with individual drugs by administering a 0.05% phenol red solution (0.15 ml/mouse). Fifteen minutes later, the mice were sacrificed by intraperitoneal injection of 20% urethane. The stomach was immediately removed and cut into several pieces in 2 ml of 0.1 N NaOH, followed by addition of 0.02 ml 20% trichloroacetic acid. The mixture was centrifuged for 10 min at 10,000 rpm, and the supernatant (0.05 ml) added to 0.5 N NaOH (0.2 ml). The absorbance of this mixed solution was measured with a spectrometer at a wavelength of 560 nm. The gastric emptying rate (%) was calculated = 100 – (A/B)×100, where A is the amount of phenol red remaining in the stomach 15 min after administration of the phenol red solution, and B is the amount of phenol red in the stomach immediately after administration of the phenol red solution.

### Fluorescence immunoassay of GLP-1 and PYY

Blood samples were collected in lavender Vacutainer tubes containing EDTA at 0 and 15 min after treatment with vehicle, cinnamaldehyde, or methyl syringate. Plasma isolated by centrifugation was collected and stored at −20°C until analysis. Plasma levels of GLP-1 and PYY were measured using a GLP-1 enzyme-linked immunosorbent assay (ELISA) kit (Linco Research, St. Charles, MO) or PYY ELISA kit (Phoenix Pharmaceuticals, Mountain View, CA, USA). To investigate the effects of TRPA1 inhibition, 0.33 mg/kg RR or 0.15 mg/kg HC-030031 were administered with vehicle (1.5% methylcellulose) or 10 mg/kg cinnamaldehyde or methyl syringate and gut hormones measured 15 min after treatment. The relative changes in PYY and GLP-1 were calculated  =  A_15 min_/A_0 min_, where A_15 min_ is the hormone level in plasma 15 min after administration of the drugs, and A_0 min_ is the hormone level in plasma at 0 min.

### Statistical analysis

All results are expressed as means ± standard error of the mean (SEM). Data analysis was performed using the GraphPad Prism software (GraphPad Software Inc., San Diego, CA, USA). The results were analyzed by one-way analysis of variance (ANOVA) and Dunnett's multiple range test.

## Results

### Effects of cinnamaldehyde and methyl syringate on food intake and remaining food in the stomach

**Figure 2:**
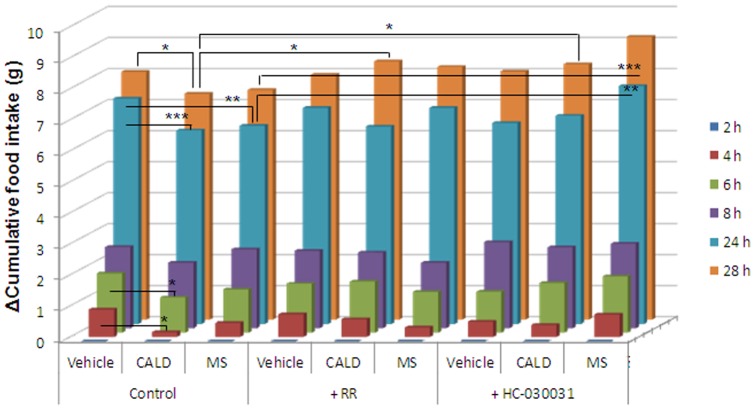
Effects of cinnamaldehyde (CALD) and methyl syringate (MS) on cumulative food intake. Vehicle (1.5% methyl cellulose), CALD, or MS were administered orally to ICR mice at a dose of 10 mg/kg after fasting in the presence or absence of the TRPA1 antagonists RR and HC-030031. Mice were allowed free access to normal food and water immediately after treatment. Changes in cumulative food intake were monitored for 28 h. Data represent means ± SEM (n = 4); *p<0.05 compared with the vehicle group by Dunnett's test.

The effects of cinnamaldehyde and methyl syringate on food intake and food remaining in the stomach were investigated. Cinnamaldehyde or methyl syringate was administered orally to ICR mice at a dose of 10 mg/kg after fasting with the 1.5% methyl cellulose vehicle used as a control. Subsequently, ICR mice were allowed *ad libitum* access to food and water. Food intake was measured at 2, 4, 6, 8, 24, and 28 h after treatment (Figure 2) and the y-axis calculated as: Δ Cumulative Food Intake (g)  =  F_2h_–F_t_, where F_2h_ is the amount of food remaining 2 h after administration of methyl cellulose, cinnamaldehyde, or methyl syringate solution and F_t_ is the amount of food remaining at each time point. Food intake rapidly increased from 0–2 h with no significant difference between the control and experimental group (1.163±0.038 g/h for 2 h, data not shown). In contrast, after 2 h, food intake decreased significantly. Therefore, cumulative food intake for 28 h was evaluated 2 h after treatment.

Cumulative food intake in cinnamaldehyde-treated mice was significantly reduced compared to the vehicle control 4 h after treatment, while that in methyl syringate-administered mice was significantly reduced 24 h after treatment. RR and HC-030031 efficiently blocked the effects of cinnamaldehyde at 28 h, but only HC-030031 inhibited the effects of methyl syringate at 24 and 28 h.

**Figure 3:**
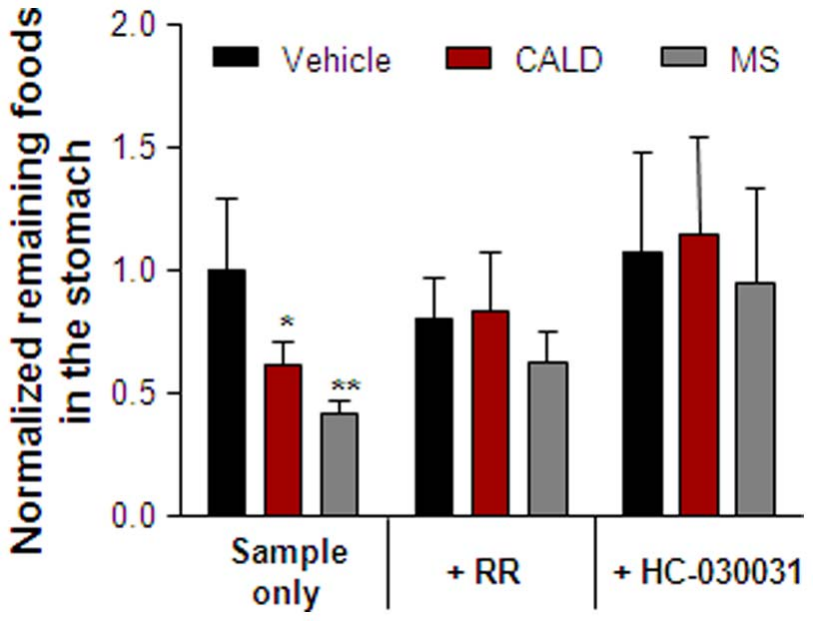
Effect of cinnamaldehyde (CALD) and methyl syringate (MS) on food remaining in the stomach. Vehicle (1.5% methyl cellulose), CALD, or MS were administered orally to ICR mice at a dose of 10 mg/kg after fasting in the presence or absence of the TRPA1 antagonists, ruthenium red (RR) and HC-030031. After 28 h, mice were sacrificed and the stomach contents of each were weighed. Columns and vertical bars represent means ± SEM (n = 4); *p<0.05 compared with the vehicle control.

Figure 3 shows the amount of remaining food in the stomach 28 h after treatment. Normalized remaining foods in the stomach were calculated as  =  R_sample_/R_vehicle,_ where R_sample_ is the amount of food remaining in the stomach 28 h after treatment with methyl cellulose, cinnamaldehyde, or methyl syringate solution and R_vehicle_ is the amount of food remaining in the stomach 28 h after treatment with the methyl cellulose vehicle only. Normalized remaining foods in the stomach of cinnamaldehyde- or methyl syringate-treated mice were significantly decreased compared to the control. Moreover, RR and HC-030031 reversed this effect in that the amount of food remaining in the stomach of cinnamaldehyde– or methyl syringate-treated mice returned to control levels.

### Effect of cinnamaldehyde and methyl syringate on gastric emptying

**Figure 4:**
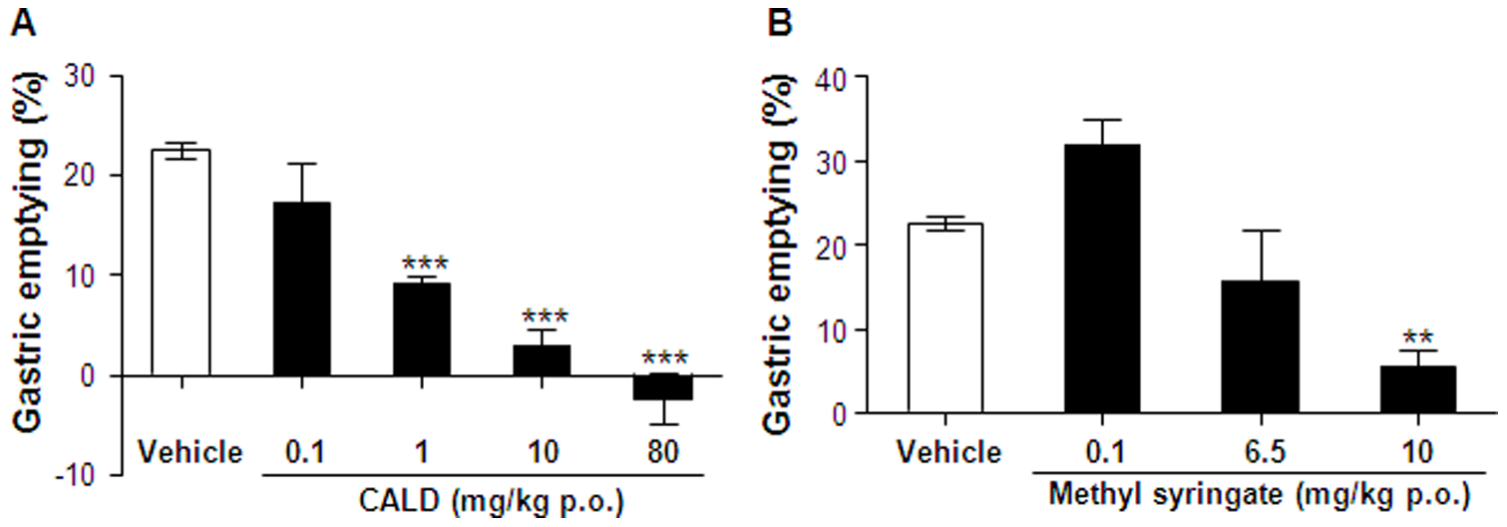
Effects of cinnamaldehyde (CALD) and methyl syringate (MS) on gastric emptying. Phenol red was administered 5 min after treatment with vehicle (1.5% methyl cellulose), CALD (0.1–80 mg/kg) and MS (0.1–10 mg/kg). Gastric emptying was evaluated by measuring the quantity of phenol red retained in the stomach 15 min after administration. Columns and vertical bars represent means ± SEM (n = 4); *p<0.05 compared with the vehicle control.

The effects of cinnamaldehyde and methyl syringate on gastric emptying were estimated in ICR mice. The body weights of ICR mice were not significantly different between the control and experimental groups (30.32±0.24 g; *p*<0.05). The effects of cinnamaldehyde (0.1–80 mg/kg) and methyl syringate (0.1–10 mg/kg) were compared with the 1.5% methyl cellulose vehicle (Figure 4). Cinnamaldehyde dose-dependently delayed gastric emptying, with a significant effect starting at 1 mg/kg and an IC_50_ value  = 4.77 mg/kg. Methyl syringate also dose-dependently delayed gastric emptying with ∼70% reduction at a dose of 10 mg/kg compared to the control.

### Inhibitory effect of RR or HC-030031 on delayed gastric emptying

**Figure 5:**
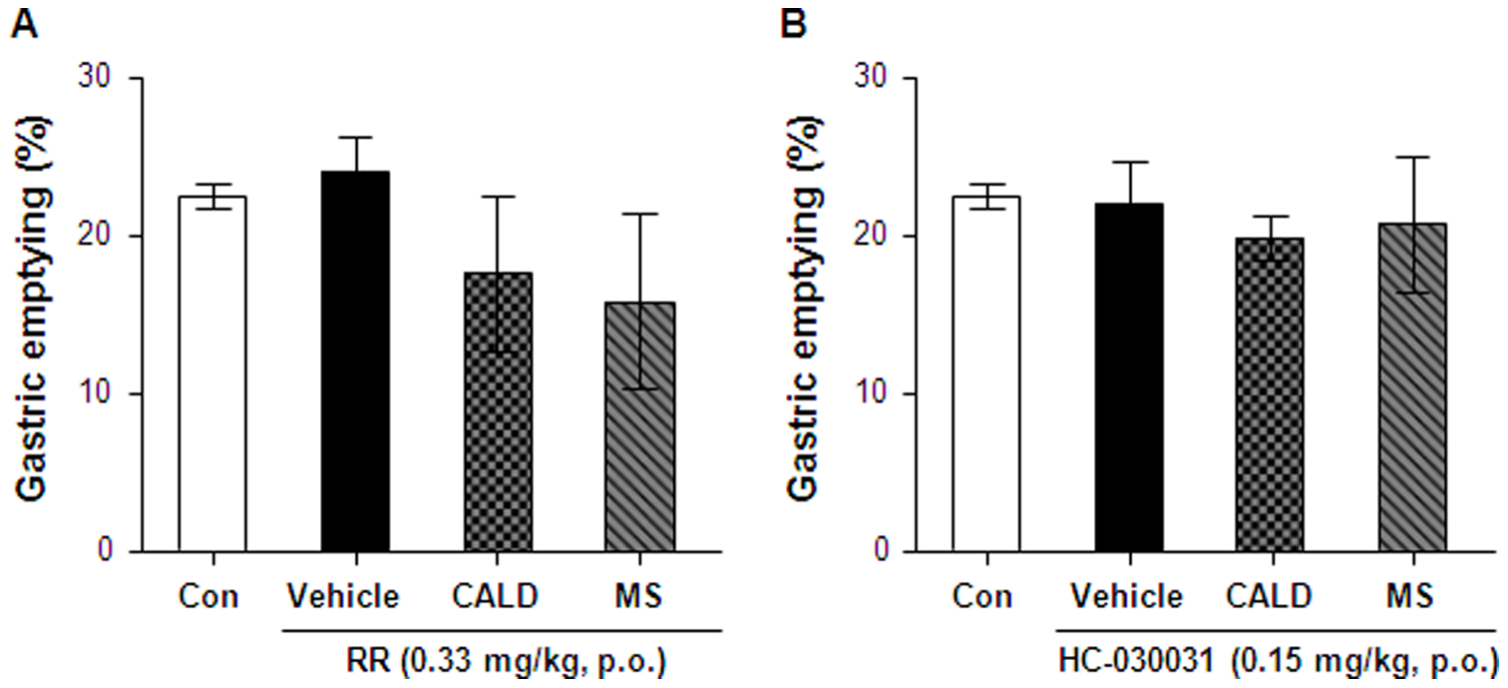
Inhibitory effect of ruthenium red (RR) or HC-030031 on gastric emptying in mice treated with cinnamaldehyde (CALD) or methyl syringate (MS). Phenol red was administered 5 min after treatment with vehicle (1.5% methyl cellulose), CALD, or MS at a dose of 10 mg/kg in the presence or absence of RR (A) and HC-030031 (B). Gastric emptying was evaluated measuring the quantity of phenol red retained in the stomach after 15 min. Columns and vertical bars represent the means ± SEM (n = 4); *p<0.05 compared with the vehicle control.

To investigate the effects of TRPA1 agonists mediated through TRPA1 channels, we examined the effects of cinnamaldehyde on mice treated with 0.33 mg/kg RR, a general cation channel blocker or 0.15 mg/kg HC-030031, a selective TRPA1 antagonist. RR and HC-030031 were administered to mice with 10 mg/kg cinnamaldehyde or methyl syringate. In the presence of RR or HC-030031, cinnamaldehyde– or methyl syringate-induced delayed gastric emptying returned to control levels (Figure 5). The effects of RR and HC-030031 on cinnamaldehyde- or methyl syringate-induced delayed gastric emptying were not significantly different and neither RR nor HC-030031 alone affected basal gastric emptying (data not shown).

### Effects of cinnamaldehyde and methyl syringate on plasma PYY and GLP-1 in mice

**Figure 6:**
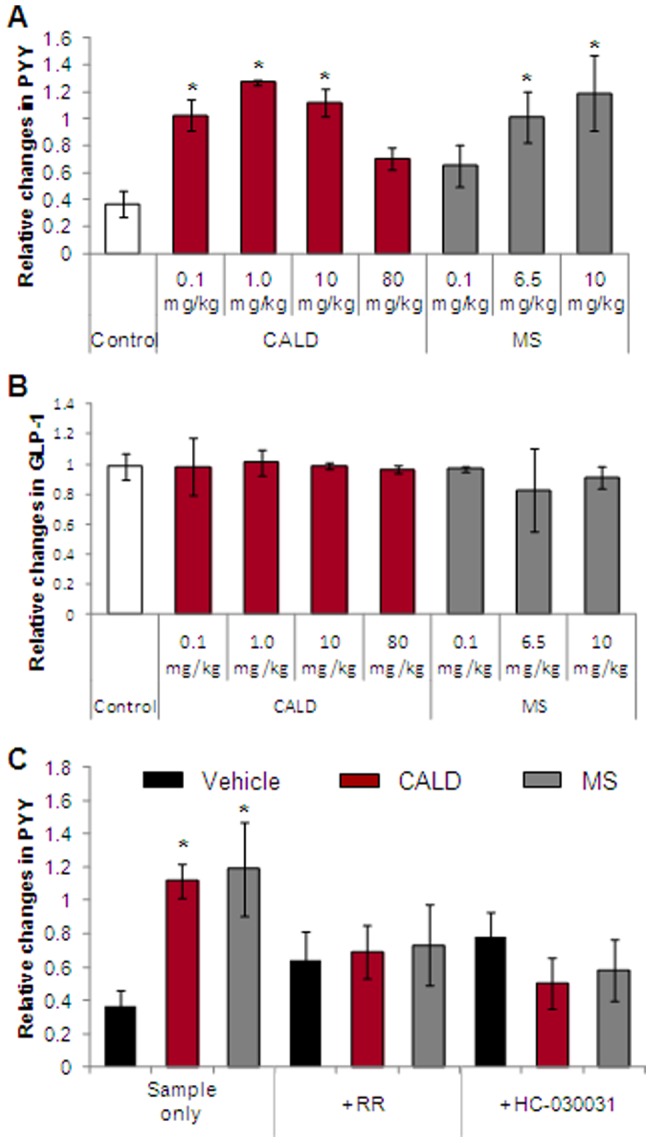
Effects of cinnamaldehyde (CALD) or methyl syringate (MS) on plasma PYY and glucagon-like peptide (GLP-1) levels. Vehicle (1.5% methyl cellulose), CALD, or MS were administered orally to ICR mice at a dose of 10 mg/kg after fasting in the presence or absence of the TRPA1 antagonists RR and HC-030031. Plasma PYY (A) or GLP-1 levels (B) upon CALD and MS treatment alone or with the addition of RR and HC-030031 (C) were evaluated at 0 min and 15 min and relative values calculated. Columns and vertical bars represent means ± SEM (n = 4); *p<0.05 compared with the vehicle control.

To investigate the involvement of cinnamaldehyde or methyl syringate on regulation of food intake, plasma PYY and GLP-1 levels were analyzed (Figure 6). Cinnamaldehyde or methyl syringate treatment increased plasma PYY levels 15 min after treatment but did not affect GLP-1 levels. We confirmed that cinnamaldehyde and methyl syringate elevated plasma PYY through a TRPA1-mediated pathway by administering RR or HC-030031. RR and HC-030031 blocked cinnamaldehyde- or methyl-syringate-induced increases in plasma PYY levels and reduced PYY in vehicle-treated animals.

## Discussion

Methyl syringate is a plant phenolic compound in *Aspergillus flavus, Aspergillus parasiticus, Betula alba, Kunzea ericoides* (kanuka) honeys, and *Leptospermum scoparium* (manuka) and has several functions as a superoxide scavenger, an effective laccase mediator, and an inhibitor of aflatoxin production [27]–[29]. However, the effects of methyl syringate on gastric functions remain unexplored. In our previous report, we demonstrated that methyl syringate from *Kalopanax pictus* activates TRPA1 and increases cytosolic Ca^2+^ concentrations in TRPA1-expressing cells [24].

TRPA1 expression has recently been identified in the stomach, small intestine, and colon within the GI tract of the rat and mouse, where it contributes to chemosensation, mechanosensation, GI motility, delayed gastric emptying, and the regulation of food intake [17], [30] In rats, the TRPA1 agonists AITC, in the range of 0.01–10 mg/kg, and cinnamaldehyde, in the range of 0.1–100 mg/kg, reduce gastric emptying. This effect is inhibited by pretreatment with the cation blocker RR (1 mg/kg). Thus, preventing TRPA1 controls gastric motility. Our results in ICR mice correlate with these data in rats. We demonstrate that cinnamaldehyde induced gastric emptying in a dose-dependent manner, while RR and HC-030031 blocked the effect of cinnamaldehyde on gastric emptying. Methyl syringate also reduced gastric emptying in a dose-dependent manner, and RR and HC-030031 suppressed delayed gastric emptying in ICR mice.

The mechanism of TRPA1-mediated gastric emptying has not been fully described. Doihara *et* *al.* (2009) suggested the serotonergic pathway as one possible mechanism. In the GI tract, more than 10 types of enteroendocrine cells exist, and TRPA1 is highly expressed in enterochromaffin (EC) cells and endocrine cells. Nutrients and non-nutrients stimulate EC cells to secrete serotonin (5-HT), which controls GI motility and enhances secretory and peristaltic reflexes, and endocrine cells to release cholecystokinin, which contributes to delayed gastric emptying and reduces food intake. The TRPA1 agonists, AITC and cinnamaldehyde, show identical effects on 5-HT secreting cells and RIN14b cells [17]. *In vitro*, AITC and cinnamaldehyde covalently bind TRPA1 and dose-dependently activate the TRPA1-mediated pathway to increase Ca^2+^ influx. Subsequently, 5-HT is secreted from EC cells and RIN14b cells. To confirm that TRPA1 mediates this response, AITC– or cinnamaldehyde-induced TRPA1 activation was assessed using TRPA1-specific siRNA and RR. The contribution of a serontonergic pathway was confirmed in male Wistar rats using the tryptophan hydroxylase (TPH) inhibitor p-chlorophenylalanine (pCPA) and the 5-HT_3_ receptor antagonist granisetron [30]. TPH-1 contributes to 5-HT secretion in EC cells via biosynthesis of 5-HT from tryptophan.

Other gastric hormones, including GLP-1 and PYY, influence food consumption and energy intake. Nutrients, such as glucose and fat, and non-nutrients, such as the neuromodulators acetylcholine, GABA, and somatostatin, stimulate GLP-1 and PYY secretion from endocrine L cells lining the small intestine into the blood to affect energy intake. Two biologically active forms of GLP-1, GLP-1 (7–37) and GLP-1 (7–36), exist with GLP-1 (7–36) being the major circulating form in humans [31]. Secreted GLP-1 binds to the GLP-1 receptor and stimulates downstream adenylyl cyclase activation and cAMP production. Moreover, peripherally injected GLP-1 induces increment *c-fos* expression in the brainstem [32]. GLP-1 also accelerates glucose-stimulated insulin secretion and suppresses glucagon secretion, gastric emptying, and food consumption. PYY has two circulating forms, PYY (1–36) and PYY (3–36) [33], [34]. The N-terminal tyrosine-proline residues from PYY (1–36) are cleaved by the enzyme dipeptidyl peptidase-4 (DPP-4) to produce the major circulating form, PYY (3–36). Many studies suggest that PYY (3–36) influences feeding via the hypothalamus [35], [36]. PYY (3–36) binding to the hypothalamic Y2 receptor activates *c-fos*, a marker of neuronal activation, and reduces food intake. This anorexic action has been demonstrated in mice intraperitoneally administered PYY (3–36) and in humans [37]. Additionally, increased GLP-1 and PYY in plasma delay gastric emptying.

We found that cinnamaldehyde and methyl syringate elevated plasma PYY levels in male ICR mice fed a liquid meal of 1.5% methylcellulose. Plasma PYY levels were increased with 0.1–1 mg/kg cinnamaldehyde, but decreased in the range from 1.0–80 mg/kg cinnamaldehyde. In contrast, cinnamaldehyde and methyl syringate had no significant effects on plasma GLP-1 levels. Theoretically, cinnamaldehyde and methyl syringate identically elevate or inhibit these hormones since PYY and GLP-1 are usually co-secreted from L cells. However, only plasma PYY was increased, which could be explained by the plasma half-life of PYY and GLP-1. Plasma PYY is elevated after 15 min, while plasma GLP-1 level is increased 10 min after food intake [38], [39]. However, the timing and magnitude of PYY and GLP-1 secretion depend on the composition and content of the meal [40]. For example, whey protein, which is absorbed faster than casein, elicited a greater GLP-1 response than casein [41]. Methyl cellulose, used in this study as a control, is a synthetic fiber derived from cellulose and had no effect on appetite. The interaction rate between cinnamaldehyde or methyl syringate in liquid form and TRPA1 in the GI tract is unknown. Also, GLP-1 showed a shorter half-life (∼2 min) than PYY (>8 min). Therefore, GLP-1 may not be detected in collected plasma.

In summary, methyl syringate, a TRPA1 activator, significantly suppressed food intake and delayed gastric emptying by elevating plasma PYY in male ICR mice. This can reduce the time necessary to perceive gastric fullness and so result in weight loss. Obesity is closely connected to type 2 diabetes, heart disease, several types of cancer, and osteoarthritis. Delayed gastric emptying is one method of reducing body weight. The current study suggests that methyl syringate, which reduced food intake, gastric emptying, and secretion of gut hormones, may contribute to weight suppression.
